# Helical organization of microtubules occurs in a minority of tunneling membrane nanotubes in normal and cancer urothelial cells

**DOI:** 10.1038/s41598-018-35370-y

**Published:** 2018-11-20

**Authors:** Nataša Resnik, Tim Prezelj, Giulia Maria Rita De Luca, Erik Manders, Roman Polishchuk, Peter Veranič, Mateja Erdani Kreft

**Affiliations:** 1 University of Ljubljana, Faculty of Medicine, Institute of Cell Biology, Ljubljana, Slovenia; 2 University of Amsterdam, Swammerdam Institute for Life Sciences, Amsterdam, The Netherlands; 3 Telethon Institute of Genetics and Medicine (TIGEM), Pozzuoli (NA), Italy

## Abstract

Tunneling membrane nanotubes (TnTs) are membrane protrusions connecting nearby or distant cells *in vitro* and *in vivo*. Functions of TnTs in cellular processes are various and rely on TnT structure, which also depends on cytoskeletal composition. In the present study, we focused on the organization of microtubules (MTs) and intermediate filaments (IFs) in TnTs of urothelial cells. We analysed TnTs of normal porcine urothelial cells, which morphologically and physiologically closely resemble normal human urothelial cells, and of cancer cells derived from invasive human urothelial neoplasm. Wide-field fluorescence, confocal and super-resolution microscopy techniques, together with image analyses and 3D reconstructions enlightened specific MT-IF organization in TnTs, and for the first time revealed that MTs and IFs co-occur in the majority of normal and cancer urothelial cell TnTs. Our findings show that in the initiation segment of TnTs, MTs are cross-linked with each other into filamentous network, however in the middle and the attaching segment of TnT, MTs can helically enwrap IFs, the phenomenon that has not been shown before within the TnTs. In this study, we assess MT-IF co-occurrence in TnTs and present evidence that such helical organization of MTs enwrapping IFs is only occurring in a minority of the TnTs. We also discuss the possible cell-biological and physiological reasons for helical organization of MTs in TnTs.

## Introduction

Tunneling membrane nanotubes (TnTs) were discovered in 2004 as thin membranous protrusions that form between cells^1^. In recent years a variety of cell types *in vitro*, and in normal and cancerous tissues *in vivo* were found to be connected by TnTs^2–4^. Since cell-to-cell communication and exchange of materials are crucial during tissue development, cell repair and survival, it is not surprising that many cells can form TnTs for exchange of different material on distances up to several hundred µm^1^. TnTs allow the trafficking of cargo from donor to acceptor cells, such as organelles, proteins, RNAs, ions, but also bacteria, viruses, prions, and nanomaterials^5–8^.

TnTs are very heterogeneous structures in terms of the length, width, longevity, the way of formation and the composition of cytoskeletal elements - actin filaments, microtubules (MTs), and intermediate filaments (IFs). Till now IFs were located in TnTs connecting cancer urothelial cells, mesothelioma cells and squamous carcinoma cells^9–11^, while evidence about MT-IF interplay in TnTs is missing. In our previous work, cytoskeleton-based classification of TnTs was proposed, namely actin filament containing TnTs were determined as type I, whereas IFs containing TnTs as type II^9^. Actin filaments are crucial for TnT formation and propagation, therefore this labelling is commonly used for TnT determination. MTs appear in TnTs of larger diameter and contribute to their stability and longevity, albeit detected in TnTs of B lymphocytes^12^, 5637 bladder cancer cells^13^, swine testicle cells^14^, RK13 kidney cells^14^, epithelial KOP cells^15^ and primary fibroblasts^15^. Both, actin filaments and MTs inside TnTs are probably important as highways on which motor proteins transport material between connected cells^16^. Presumably, IFs were overlooked in TnTs studies because of their established role in the mechanical support of cells, and yet unknown role in the cargo transport. For the effective transport of material through TnT and for the stability of TnT, the organization and position of all three cytoskeletal elements is expected to be important.

In this study, we focused on MT and IF composition and their organization in TnTs of urinary bladder normal and cancer urothelial cells. In this regard, we used normal porcine urothelial (NPU) cells, which are accepted as an equivalent to human normal urothelial cells on the basis of their molecular, ultrastructural and physiological characteristics^17–21^. As a counterpart human cancer urothelial T24 cells of high-grade muscle invasive urothelial carcinoma were used. We provided new insights into cytoskeletal structure of TnTs, particularly MT-IF co-occurrence in TnTs were assessed. We observed the helical organization of MTs enwrapping IFs, the phenomenon that has not been seen before within the TnTs. For that purpose, we provided the comparative results obtained by various microscopes, image analyses and 3D reconstructions.

## Results

### Cytoskeletal characterization of TnTs

**Figure 1:**
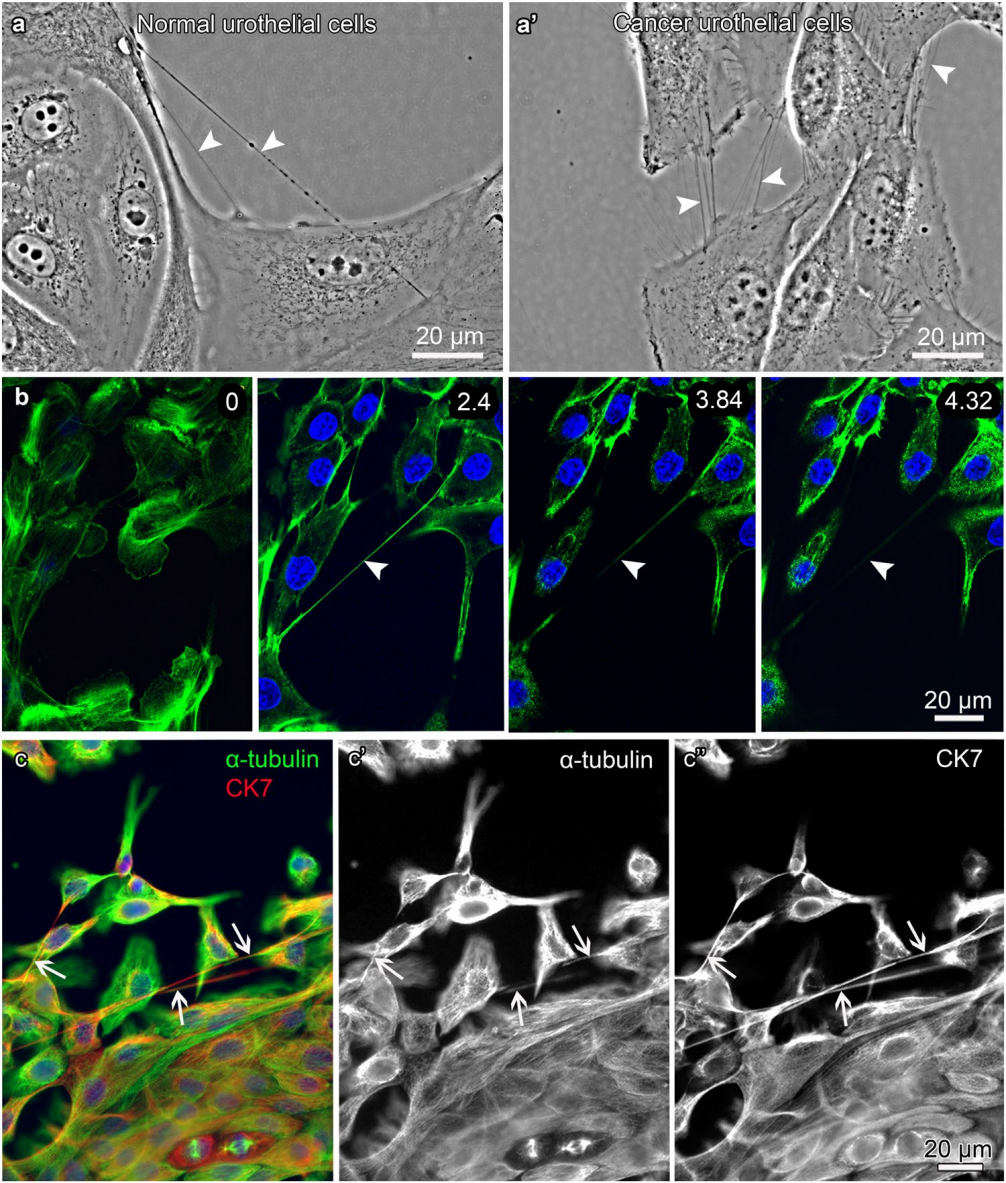
Characterization of TnTs. (**a**-**a′**) Arrowheads denote TnTs of living/unfixed normal and cancer urothelial cells. (**b**) Actin filaments (F-actin, green) in normal urothelial cells. Panel b shows four sequential optical sections with relative z-positions (in µm) and indicates that TnT (arrowheads) does not adhere to the substratum. (**c**-**c″**) Arrows denote TnTs of normal urothelial cells with MTs (α-tubulin, green) and IFs (CK7, red) co-labelling, on merged (**c**) and separate images (**c′**-**c″**). Images were obtained with phase-contrast and wide-field fluorescence microscope with ApoTome device. Panel (b) was performed after paraformaldehyde fixation and (**c**) after methanol fixation, both of normal urothelial cells.

**Figure 2:**
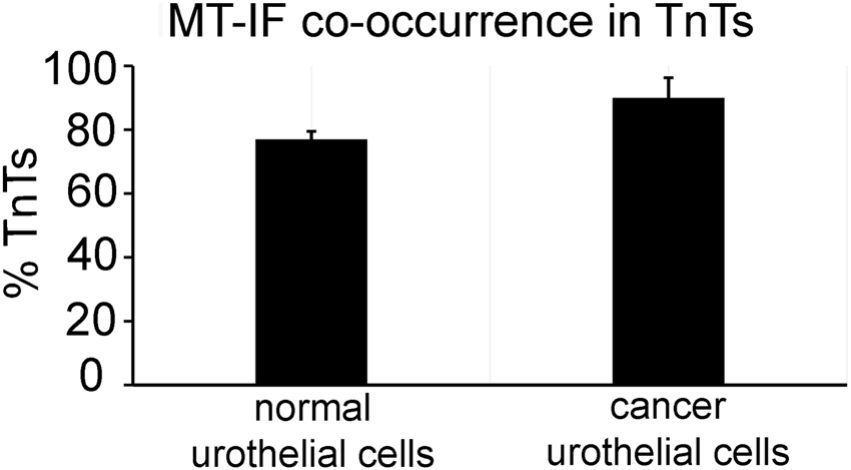
The quantification of TnTs with MT-IF co-occurrence in normal and cancer urothelial cells. Data are presented as mean ± standard error of the mean from five independent imunolabellings and 50 TnTs were altogether analysed for each cell type. *p* = 0.2.

Normal urothelial cells formed TnTs between nearby and distant cells (Fig. 1a,b,c-c″), similarly as cancer urothelial cells (Fig. 1a′). We grew cells in physiological medium at seeding densities 5 × 10^3^, 5 × 10^4^, 1 × 10^5^ and 2 × 10^5^ cells/cm^2^ for two days to assess the highest number of TnTs (Supplementary Fig. S1). Counting TnTs per 1 cm^2^ revealed that normal urothelial cells form the highest number of TnTs which was 2.2 ± 0.9 at the density 1 × 10^5^ cells/cm^2^ and cancer urothelial cells 30.5 ± 8.3 TnTs at 5 × 10^3^ cells/cm^2^ (Supplementary Fig. S1). TnTs included actin filaments (Fig. 1, panel b) and they did not adhere to the substratum (Fig. 1, sequential optical sections in panel b), satisfying the main criteria for TnT identification. Double immunolabelling proved MT and IF co-occurrence in TnTs (Fig. 1c-c″). The quantification of proportional distribution of both cytoskeletal elements demonstrated that the majority of normal and cancer urothelial TnTs is composed of MTs and IFs, that is in 77.0 ± 2.5% of TnTs connecting normal urothelial cells and in 90.0 ± 6.3% of TnTs connecting cancer urothelial cells (Fig. 2).

### MT-IF interplay in TnTs

**Figure 3:**
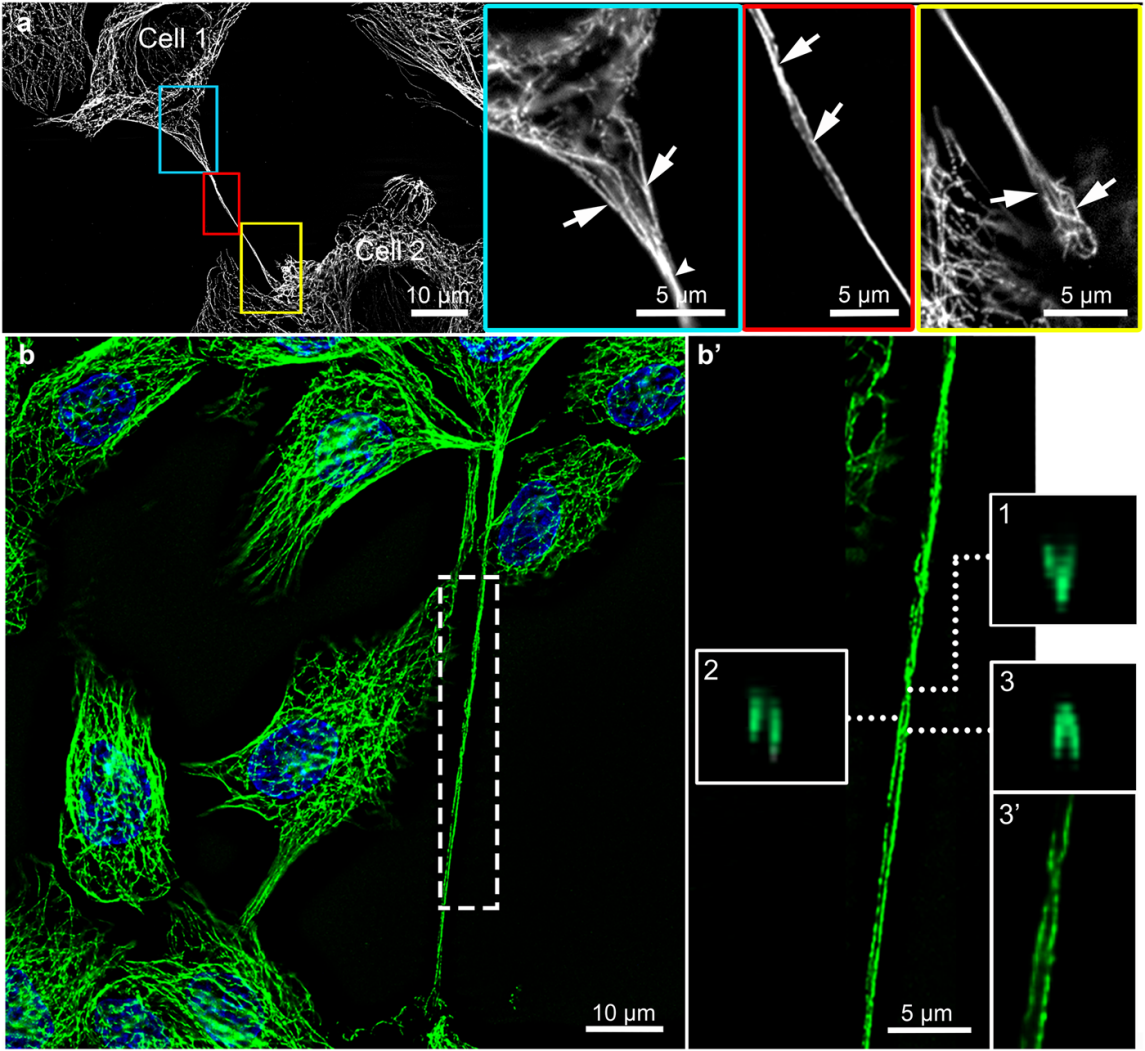
Organization of MTs in TnTs as revealed with wide-field fluorescence microscopy after deconvolution of optical sections. (**a**) The initiation segment (in blue frame), middle segment (in red frame) and the attaching segment (in yellow frame) of TnT with MTs (α-tubulin) are highlighted. The initiation segment shows filamentous network of MTs (arrows), which begin to compact (arrowhead) toward the middle segment (blue framed inset). The middle segment of TnT shows MTs twisted around each other (arrows in red framed inset). At the attaching segment (yellow framed inset) MTs are filamentous again (arrows). (**b**) Maximum intensity projection (x, y) of deconvolved fluorescence images. Dashed frame is enlarged in (**b′**). Three successive intersections of x-z projections show (**1**) the first MT-crossing, (**2**) parallel MTs and (**3**) second MT-crossing. (**3′**) 3D close-up visualization of (**3**) the region of MT-crossing. Dotted lines point to the x-z intersections 1–3 (×3 enlarged images of the image **b′**). Image (**a**) was performed after formalin/glutaraldehyde fixation of cancer urothelial cells and (**b**) after methanol fixation of normal urothelial cells.

**Figure 4:**
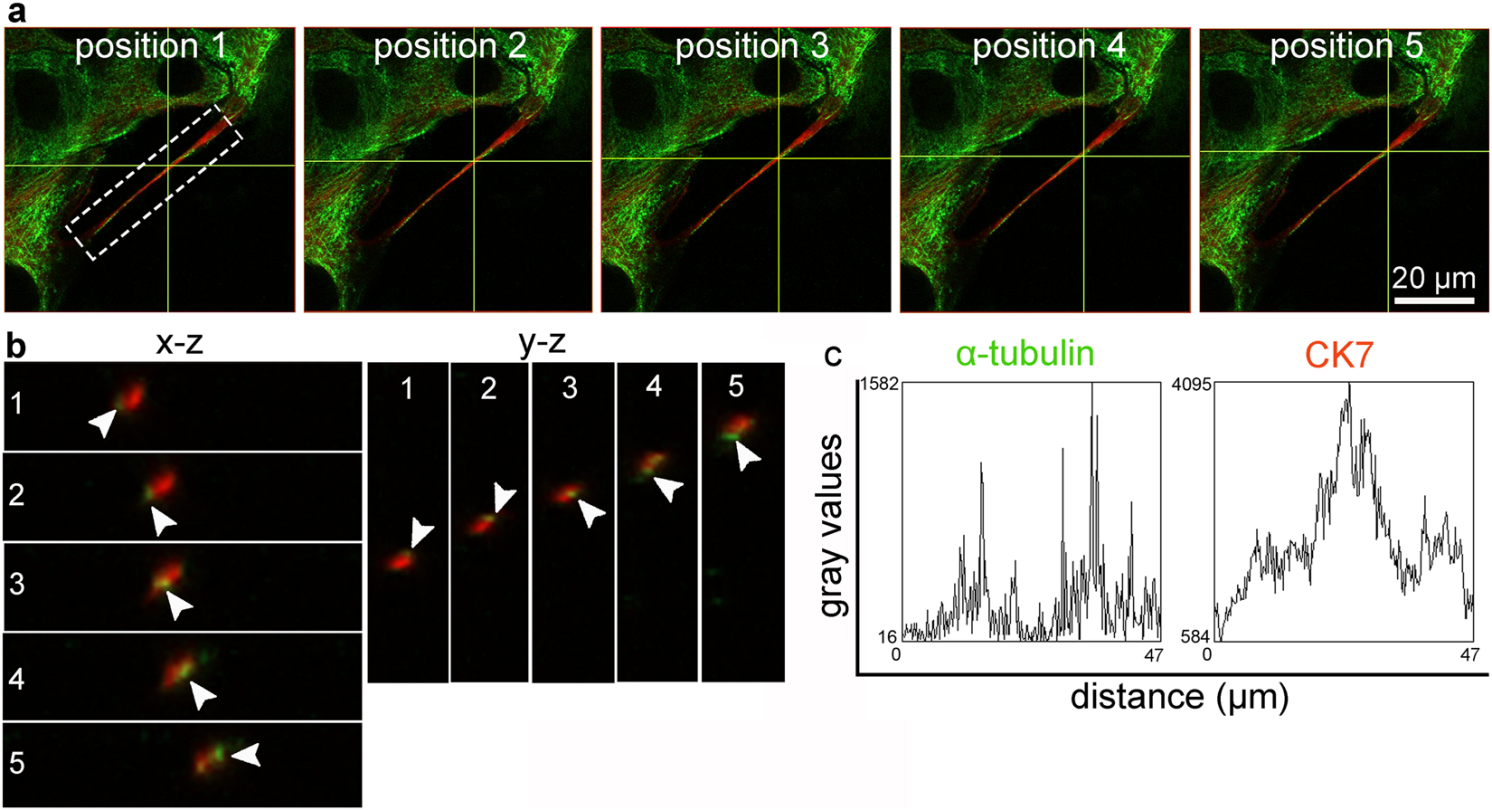
MTs are twisting around IFs in TnT as observed with confocal microscopy. (**a**) Positions 1–5 correspond to (**b**) x-z and y-z projections and show that MTs (α-tubulin, green) rotate around IF core (CK7, red). Note the rotation of MTs (arrowheads) in x-z and in y-z. (**c**) Gray values of MTs (α-tubulin) and IFs (CK7) fluorescence intensity along TnT are plotted. Gray values of α-tubulin reach point 0 between two peaks, which correspond to one twist of MTs around IFs. Presented normal urothelial cells were methanol fixed.

**Figure 5:**
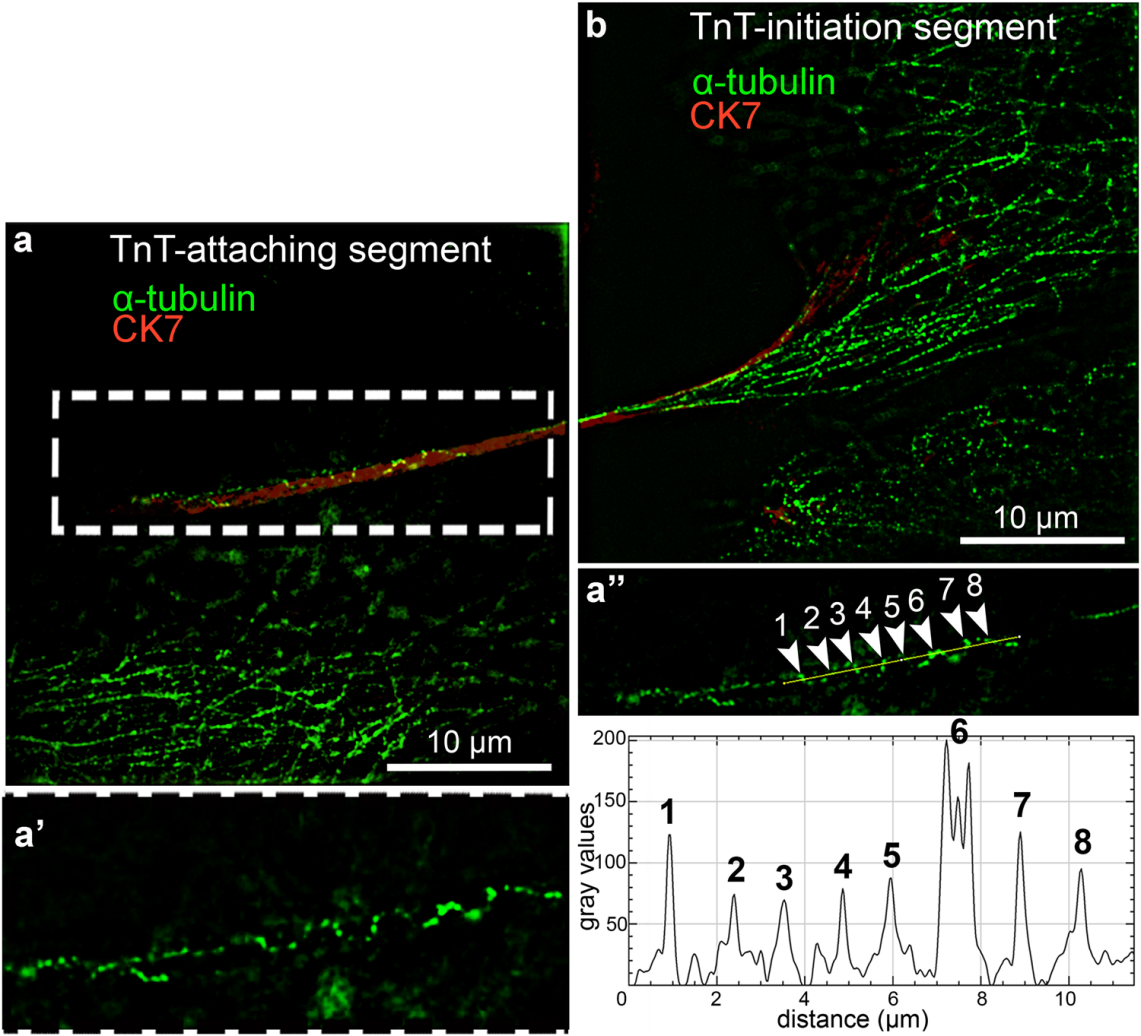
Helical organization of MTs in TnT as observed with 3D-SIM microscopy. (**a**,**b**) Maximal intensity profile of x-y projections of MT signal (α-tubulin, green) and IF signal (CK7, red) at the initiation segment and the attaching segment of TnT. Dashed white region is × 1.5 enlarged in (**a′**) and represents x-y projection with helical MT organization (**a″**). Gray values on graph correspond to the fluorescence intensity signal of MTs (α-tubulin) along yellow line. Eight white arrowheads correspond to the eight peaks on graph and indicate four MT twists. Presented normal urothelial cells were fixed with methanol.

**Figure 6:**
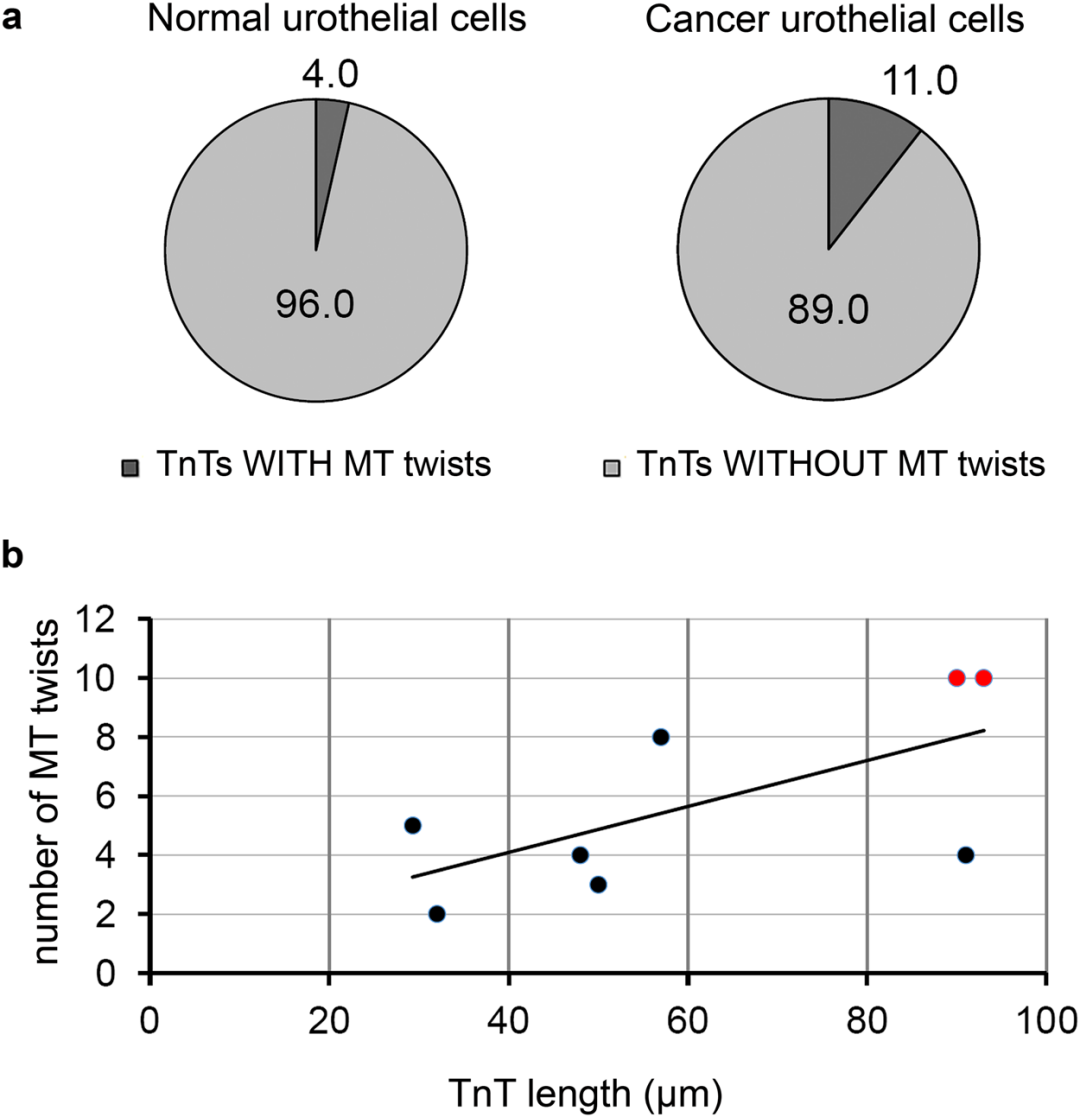
Quantification of MT twists in TnTs. (**a**) Proportion of all TnTs with or without MT twists in normal and cancer urothelial cells. (**b**) Number of MT twists per TnT length. Only TnTs with MT twists were analysed. Black dots denote individual TnT of cancer urothelial cells and red dots individual TnT of normal urothelial cells. In each cell type 50 TnTs were analysed (n = 50).

Further we studied organization of MTs and IFs through TnTs and found that at the initiation segment of the TnT, MTs were cross-linked with each other into filamentous network (Fig. 3a, blue frame). This MT network started to compact in the middle of TnT (Fig. 3a, red frame, arrows), and reorganized into twisted structure (Figs 3 and 4), which could be present also at the attaching segment (Fig. 5). Such twisted organization is evident from x-y intersections of TnT (Fig. 3b,b′ frames 1–3) and 3D reconstruction (Fig. 3b′, frame 3′). Further double immunolabelling of α-tubulin and CK7 showed that MTs helically enwrap the core of IFs (Figs 4 and 5). Twists of MTs around IFs are less (e.g. one turn, Fig. 4c) or more (e.g. four turns, Fig. 5a′′) frequent. Figure 4 shows helical arrangement of MTs around IF core in five successive x-z and x-y projections of MTs and IFs in the middle segment of TnT. Figure 5 shows MT twisting in the attaching segment of TnT. Eight peaks on graph correspond to α-tubulin fluorescence intensities (Fig. 5a′′), indicating four turns of MTs. TnTs of urothelial cells had MT twists located in the middle and/or attaching segment of TnTs. The number of TnTs with twisted MTs was higher in cancer than in normal urothelial cells, 11% *vs* 4% (n = 50), respectively (Fig. 6a). Altogether, urothelial TnTs have 2–10 twists per TnT (Fig. 6b).

## Discussion

Here we report for the first time that MTs can be helically organized in TnTs. It is important to note that TnT growth was not provoked by stress, but the cells formed TnTs in physiological conditions. Although presence of actin filaments is a crucial criterion for TnT determination, in this study we were focused on MTs and IFs. In general, TnTs are diverse in lengths and thickness, and display a pronounced sensitivity to light excitation, mechanical stress and chemical fixation, leading to the rupture of many TnTs between cells^22^. In this study, we provide new insights into MT and IF organization within TnTs of normal and cancer urothelial cells. The key results of double immunolabelling and analyses of images are a) the proof of MT organization along TnTs and b) quantification of MT and IF co-occurrence in TnTs. Our analysis is the first to reveal that MTs are twisted around each other, as well as around IFs in TnTs, performing the helical organization. This phenomenon has not been described in other cells or even in other regions of the cells. Researchers usually screen for the presence of α- or β-tubulin inside TnT, or acetylated and tyrosinated tubulin^2,14,15^. Although inspection was made with super-resolution microscope, Osteikoetxea-Molnar *et al*. did not report about particular MT organization^12^. The nearest similarity to helical organization of MTs we found in publication of Leijnse and co-workers, studying actin filaments^23^. They revealed that actin is helically organized inside filopodia of HEK293 cells. Actin could simultaneously rotate and helically bend within cellular membrane tubes obtained by elongation of pre-existing filopodia. Such helical organization was involved in force generation.

We would like to emphasize that typical parallel MT organization in TnT is prevailing and the twisted one is rare, corresponding to only 4–11% of urothelial TnTs. In structural studies made on isolated MTs with cryo-electron microscopy, a variety of MT assemblies such as rings, ribbons, hoops, sheets were found^24^. They speculated that such assemblies might have important biological functions during cellular processes where the dynamics of MTs is needed. Surprising discovery of a twisted MT organization can for example support the elongation and enable the longevity of TnTs. In the example of sperm development, MTs wind helically around the flagellar axoneme, where they are thought to help localize the mitochondria in the sperm tail^25^.

Non-canonical MT organization could be governed by posttranslational acetylation of α-tubulin that rules protofilament number of MTs^26,27^. Additionally, transitions between different protofilament number could be observed within a single MT^26^. Moreover, when MTs are growing during TnT protrusion, MTs can occasionally switch into energetically unfavorable configurations as Chretien and Fuller demonstrated for growing calf brain tubulins^26^.

The interplay between MTs and IFs is complex and mutual^28^ and explains why the majority of urothelial TnTs is composed of both MTs and IFs, specifically cytokeratin 7. Nieuwenhuizen and co-workers showed that vimentin runs parallel with MTs in HUVEC endothelial cells, but not in 3T3 fibroblasts, showing that structural interplay between two cytoskeletal networks is cell-type specific^29^.

A hypothetical explanation of helical organization of MTs enwrapping the IFs in TnTs might lie in different elastic properties of MTs and IFs. It is known that IFs have a considerable ability to stretch while MTs are known to be very rigid^30,31^. The length of TnTs in connected motile cells can change considerably and the length of IF inside TnTs can adjust by their intrinsic elasticity. Since the MTs cannot stretch or shrink, the surplus length of MTs in approaching cells might cause helical organization around IFs, and when cells are moving apart such organization of MTs can enable prolongation of TnTs without the need of MT growth.

In conclusion, our findings illustrate specific MT organization as well as specific MT-IF interplay inside the TnTs of urothelial cells. The information provided by this approach is unique and we expect that further exploration of the role of MT organization in future functional studies may broaden our understanding of their organization and function in cell-to-cell communication via TnTs.

## Materials and Methods

### Cell culturing

Cultures of normal urothelial cells were established from normal porcine urinary bladders as described previously^17,32^. The experiments were approved by the Veterinary Administration of the Slovenian Ministry of Agriculture and Forestry in compliance with the Animal Health Protection Act and the Instructions for Granting Permits for Animal Experimentation for Scientific Purposes. Normal porcine urothelial cells from VII to XII passages were cultured in UroM medium, which consisted of equal parts of MCDB153 medium and Advanced-Dulbecco’s modified essential medium (Thermo Fisher Scientific, Invitrogen, Austria), and was supplemented with 0.1 mM phosphoethanolamine 15 μg/ml adenine 0.5 μg/ml hydrocortisone 5 μg/ml insulin), 4 mM glutamax (Thermo Fisher Scientific), 0.9 mM calcium, 2.5% FBS (Thermo Fisher Scientific), 100 μg/ml streptomycin, and 100 U/ml penicillin^33^. T24 cell line of cancer cells originated from human invasive urothelial neoplasm (ATTC, Manassas, VA, USA) was cultured in Advanced-Dulbecco’s modified essential medium and medium F12 (1:1), 5% FBS, 100 μg/ml streptomycin, and 100 U/ml penicillin. The seeding densities of normal and cancer urothelial cells were 5 × 10^3^, 5 × 10^4^, 1 × 10^5^ and 2 × 10^5^ cells/cm^2^. Seeding densities 5 × 10^3^ cells/cm^2^ of cancer urothelial cells and 1 × 10^5^ cells/cm^2^ of normal urothelial cells were used as densities with the highest number of TnTs on the second day of growth. Quantification was made in three independent experiments, each made in triplicates. Cells were grown on plastic dishes or glass coverslips at 37 °C in a humidified atmosphere and 5% CO_2_. The immunolabelling was performed on the second day of cultivation. Chemicals were purchased from Sigma-Aldrich (Taufkirchen, Germany), unless otherwise stated.

### Labelling of cytoskeletal elements

For F-actin staining, urothelial cells were fixed in 4% formaldehyde in PBS for 10 min at 22 °C, washed in PBS and labelled with 16.7 µg/ml phalloidin-FITC (Sigma-Aldrich) for 30 min at 22 °C. For double immunolabelling of α-tubulin – the subunit of MTs and cytokeratin 7 (CK7) – from the keratin subfamily of IFs, urothelial cells were fixed in 100% ice-cold methanol for 5 min washed in PBS and left in blocking buffer (1% bovine serum albumin and 10% goat serum in PBS) for 1 h. Afterwards the cells were incubated with rabbit polyclonal anti-human α-tubulin (Abcam 15246, 1:20) and mouse monoclonal anti-human CK7 (M7018 Dako, 1:20) primary antibodies for 1 h at 37 °C. For negative controls, the incubation with primary antibody was omitted. After washing with PBS, goat anti-rabbit AlexaFluor 488 (A11008, Thermo Fisher Scientific, 1:400) and goat anti-mouse AlexaFluor 555 (A21422, Thermo Fisher Scientific, 1:400) secondary antibodies were applied for 1 h at 37 °C. After labelling, coverslips were washed in PBS and embedded in Vectashield with 2-(4-Amidinophenyl)-1H-indole-6-carboxamidine (DAPI, Vector Laboratories, Peterborough, UK). When labelling α-tubulin alone, the same procedure was used. Alternatively, double immunolabelling was performed with 3.7% formalin (made from 37% formaldehyde, Acros Organics, Geel, Belgium), 0.1% glutaraldehyde (Serva) and 0.2% Triton X-100 diluted in PEM buffer for 5 min at 37 °C, indicated as formalin/glutaraldehyde/PEM fixation^34,35^. Cells were washed with PBS and post-fixed in 3.7% formalin in PBS. Washing, blocking and incubation with primary and secondary antibodies were the same as above. Co-occurrence of MTs and IFs in TnTs were analysed on images that were acquired after double immunolabelling.

### Imaging of TnTs and cytoskeletal elements

Living cells were examined with the wide-field fluorescence microscope AxioImager. Z1 (Zeiss) using water immersion objective (63×/NA 0.95) with phase-contrast. Fixed and labelled cells were examined with Zeiss LSM 510 confocal microscope (oil objective 100×/1.4 NA) and AxioImager. Z1 (63×/NA 1.40 oil immersion objective) with an ApoTome device for optical sectioning. Those images were deconvolved with Huygens Professional version 16.10 (Scientific Volume Imaging, The Netherlands, http:/svi.nl), using the CMLE algorithm, with SNR = 40 and 100 iterations. Super-resolution microscopy was performed using three dimensional structured illumination microscopy (3D-SIM) with a Nikon N-SIM system, set up on Nikon A1 microscope (100×/NA 1.49 oil immersion objective) The settings of the acquisition and the reconstruction of the SIM datasets were tuned in order to minimize the artefacts. Several reconstructions parameters, as available in the N-SIM microscope, were tested and set at the values that provided the more robust results. Image analysis and 3D reconstructions were made with ImageJ program (NIH). For the statistical analysis, the length of individual TnT from initiation to attaching segment was measured and number of MT twists was carried on deconvolved images.

### Statistical analyses

Statistical differences were determined by applying a two tailed Student *t*-test. Data are presented as mean ± standard error of the mean. The level of statistical significance was set at p < 0.05 for all tests.

## Electronic supplementary material

Supplementary Figure S1
