# Supplementary material for: Helical organization of microtubules occurs in a minority of tunneling membrane nanotubes in normal and cancer urothelial cells
Source: Sci Rep. 2018 Nov 20;8:17133. doi: 10.1038/s41598-018-35370-y (PMC6244236; doi:10.1038/s41598-018-35370-y)
Supplement: Supplementary file 1 — Supplementary Figure S1 [file 41598_2018_35370_MOESM1_ESM.docx]

**SUPPLEMENTARY INFORMATION**

**Helical organization of microtubules occurs in a minority of tunneling membrane nanotubes in normal and cancer urothelial cells**

Nataša Resnik^1^, Tim Prezelj^1^, Giulia De Luca^2^, Erik Manders^2^, Roman Polishchuk^3^, Peter Veranič^1^, Mateja Erdani Kreft^1^*

^1^University of Ljubljana, Faculty of Medicine, Institute of Cell Biology, Ljubljana, Slovenia

^2^University of Amsterdam, Swammerdam Institute for Life Sciences, The Netherlands

^3^Telethon Institute of Genetics and Medicine (TIGEM), Pozzuoli (NA), Italy

*Corresponding author:

M. E. Kreft,

Institute of Cell Biology, Faculty of Medicine, University of Ljubljana, Vrazov trg 2, SI-1000

Ljubljana, Slovenia

E-mail: mateja.erdani@mf.uni-lj.si

Tel.: +386 1 543 7685

Fax: +386 1 543 7681

**Supplementary Figure 1:**


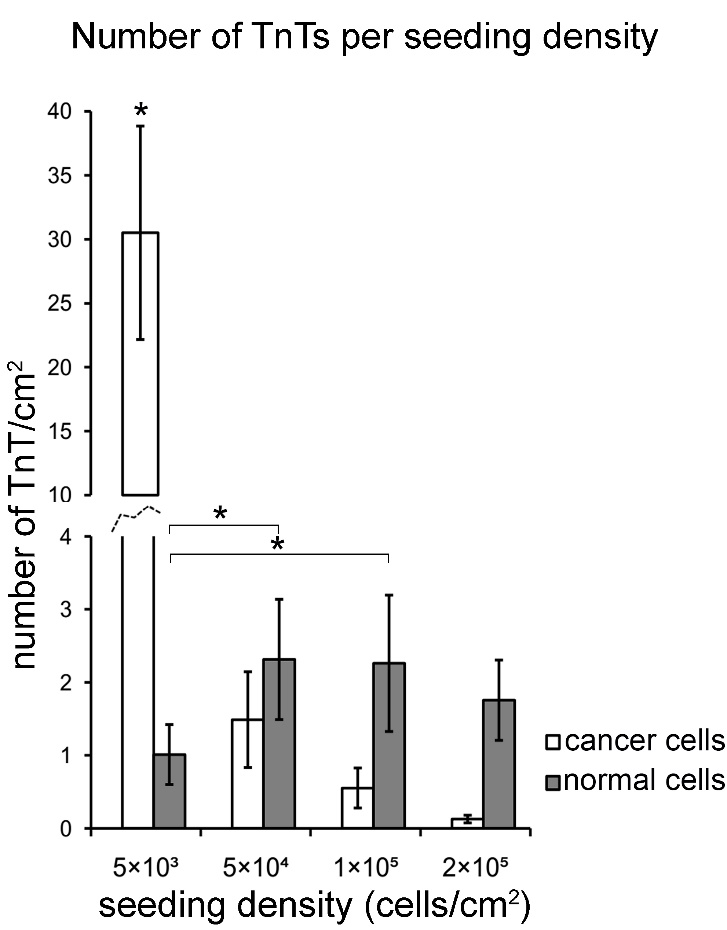


**Supplementary Figure S1: Dependence of seeding density on the number of TnTs in urothelial cells.**

The number of TnTs was examined in living cells on the second day of growth using phase-contrast microscope. Graph shows that cancer urothelial cells form the highest number of TnTs when cultured with density 5×10^3^ cells/cm^2^ and normal urothelial cells when cultured with density 1×10^5^ cells/cm^2^. Quantification was made in three independent experiments, each made in triplicates. Presented data are mean ± standard error of the mean. * *p* < 0.05.
